# Health taxes for healthier lives: an opportunity for all governments

**DOI:** 10.1136/bmjgh-2023-013761

**Published:** 2023-10-19

**Authors:** Tedros Adhanom Ghebreyesus, Helen Clark

**Affiliations:** 1 World Health Organisation, Organisation mondiale de la Sante, Geneve, Switzerland; 2 Chair of the Board, The Alliance for Health Policy and Systems Research, WHO, Geneva, Switzerland

**Keywords:** Health policies and all other topics

**Summary box**

- Health taxes are one of the most effective ways to reduce the consumption of unhealthy products helping also reduce the deaths of the more than 40 million people who die annually from non-communicable diseases
- Yet, the implementation of health taxes globally remains vastly underutilised. For example, WHO’s 2021 Report on the Global Tobacco Epidemic finds that only 13% of the world’s population is protected by tobacco taxes at best-practice levels
- WHO and the Alliance are supporting local researchers around the world to document policies and processes to advance health taxes
- Governments around the world are missing an efficient and cost-effective opportunity to improve health, raise revenue, invest in equity and save lives

One of the WHO’s highest priorities is to support countries to promote health and prevent disease by addressing its root causes.^1^ Compelling evidence demonstrates that one of the most effective ways to do this is to use health taxes to reduce the consumption of unhealthy products, ideally as part of a strategic public health approach.^2–4^ Health taxes can help reduce the deaths of the more than 40 million people who die annually from non-communicable diseases caused largely by a combination of tobacco, alcohol and unhealthy diets.^3^

Examples from around the world illustrate the impact that health taxes can have. Between 2005 and 2011, the price of cigarettes in Turkey increased by 195%; during the same period, cigarette sales dropped by 15.5% and government revenues increased by 124%.^5^ In the Philippines, tobacco use dropped by one-third between 2009 and 2021, following the introduction of a strong tobacco tax.^6^ In Russia, alcohol taxes, as part of a comprehensive alcohol control policy, contributed to an almost 50% decline in recorded consumption between 2003 and 2016.^7^ In Mexico, following the introduction of a tax on sugary drinks in 2014, consumption dropped by 5.5% in that year and 9.7% in 2015.^8^ Other countries, as diverse as Brazil, Finland, South Africa and Thailand, have shown positive health outcomes as the result of targeted tax policies.^3 4^

There are encouraging signs that the use of health taxes globally is building. In the past 5 years, more than two-thirds of WHO’s Member States have either introduced or increased taxes on at least one health-harming product, such as tobacco, alcohol or sugary drinks.^9^ For example, with support from WHO, Timor Leste last year increased its tax on tobacco fivefold, increased tax on alcohol, and introduced new taxes on sugar and sugary drinks.^10^ There is also increasing interest for health taxes on salt and other products.^11 12^

However, the implementation of health taxes globally remains vastly underutilised. For example, WHO’s 2021 Report on the Global Tobacco Epidemic finds that only 13% of the world’s population is protected by tobacco taxes at best-practice levels.^13^ Taxes on alcohol and sugary drinks are even lower.^14 15^ Despite overwhelming evidence, governments around the world are missing an efficient and cost-effective opportunity to improve health, raise revenue, invest in equity and save lives.

WHO recognises that increasing any tax is a political challenge. Countries that have introduced or increased health taxes have faced opposition from entrenched commercial interests and powerful industry lobbies. Lawmakers may also be shy of raising taxes that affect people or industries in their constituencies. It can also be difficult to convince Ministries of Finance that long-term health benefits outweigh any potential short-term economic losses. Understanding the national context—the politics, the process and power—is critical. This is why WHO is supporting local researchers around the world to document policies and processes to advance health taxes.^16^

The global economy is confronting multiple challenges. Health taxes can be an important part of the solution. As WHO’s Council on the Economics of Health for All has found, health taxes can help governments raise revenues to invest in health, education and other social priorities. These revenues can and should be invested for the poorest as a win-win for health equity and contribute to financing the achievement of the health targets of the Sustainable Development Goals. This special issue of *BMJ Global Health* is an important contribution to the public policy debate on health taxes, which if used correctly, are a powerful tool for preventing disease, saving lives, and building sustainable societies and economies.

## Data Availability

All data relevant to the study are included in the commentary.
